# Laparoscopic treatment of intestinal obstruction due to a vitelline vascular remnant and simultaneous appendicitis: a case report

**DOI:** 10.1186/s40792-018-0515-3

**Published:** 2018-08-30

**Authors:** Kenjiro Date, Taro Yokota, Naoki Maehara

**Affiliations:** Department of Surgery, Fujimoto General Hospital, 17-1 Hayasuzu-cho, Miyakonojo, Miyazaki 885-0055 Japan

**Keywords:** Vitelline vascular remnant, Intestinal obstruction, Appendicitis, Laparoscopic surgery

## Abstract

**Background:**

The presence of a vitelline vascular remnant is rare, and definitive preoperative diagnosis is difficult. We herein describe a case of intestinal obstruction caused by a vitelline vascular remnant with mild chronic appendicitis successfully diagnosed and treated with laparoscopic surgery.

**Case presentation:**

A 14-year-old male was admitted to our hospital with sudden-onset right lower abdominal pain and vomiting. A blood test on admission revealed slight leukocytosis. Computed tomography scan showed that the appendiceal wall was enhanced and thickened. Although the ileum was slightly dilated and ascites was present at the recto-vesical pouch, these were thought to be inflammatory changes secondary to appendicitis. Laparoscopic surgery was performed using three trocars. Strangulated small bowel obstruction caused by a band connecting the right medial umbilical fold to the ileal mesentery was found intraoperatively. After reduction, neither ischemic change of the small intestine nor Meckel’s diverticulum was detected. The appendix was slightly inflamed, and serous ascites was present at the recto-vesical pouch; therefore, appendectomy was also performed. The patient was discharged on postoperative day 4 without complications. Pathological examination revealed that the band consisted of blood vessels, and it was diagnosed as a vitelline vascular remnant. The appendix included fecal stones and showed chronic inflammatory change histologically; the patient was thus diagnosed with chronic appendicitis.

**Conclusions:**

Definitive preoperative diagnosis of a vitelline vascular remnant, especially with coexisting appendicitis, might be difficult. Laparoscopic surgery might be useful for patients who demonstrate unusual symptoms because it allows for simultaneous diagnosis and treatment.

## Background

A vitelline duct remnant is a rare condition that is reportedly present in approximately 2% of the general population [1, 2], and a vitelline vascular remnant is even more rare [3]. The definitive diagnosis of a vitelline duct or vascular remnant is often difficult, and its presence is an infrequent cause of intestinal obstruction. Surgeons sometimes encounter a vitelline duct or vascular remnant during emergency laparotomy for intestinal obstruction [4, 5].

Despite recent improvements in imaging studies, achieving an accurate preoperative diagnosis of acute abdomen remains difficult. Laparoscopic surgery, which allows for simultaneous exploration of the abdominal cavity and definitive diagnosis, has become a popular technique for patients with acute abdomen. However, reports of the simultaneous occurrence of intestinal obstruction caused by a vitelline vascular remnant and appendicitis treated with laparoscopic surgery are extremely rare. We herein report a case of intestinal obstruction caused by a vitelline vascular remnant with chronic appendicitis successfully treated with laparoscopic surgery.

## Case presentation

**Fig. 1:**
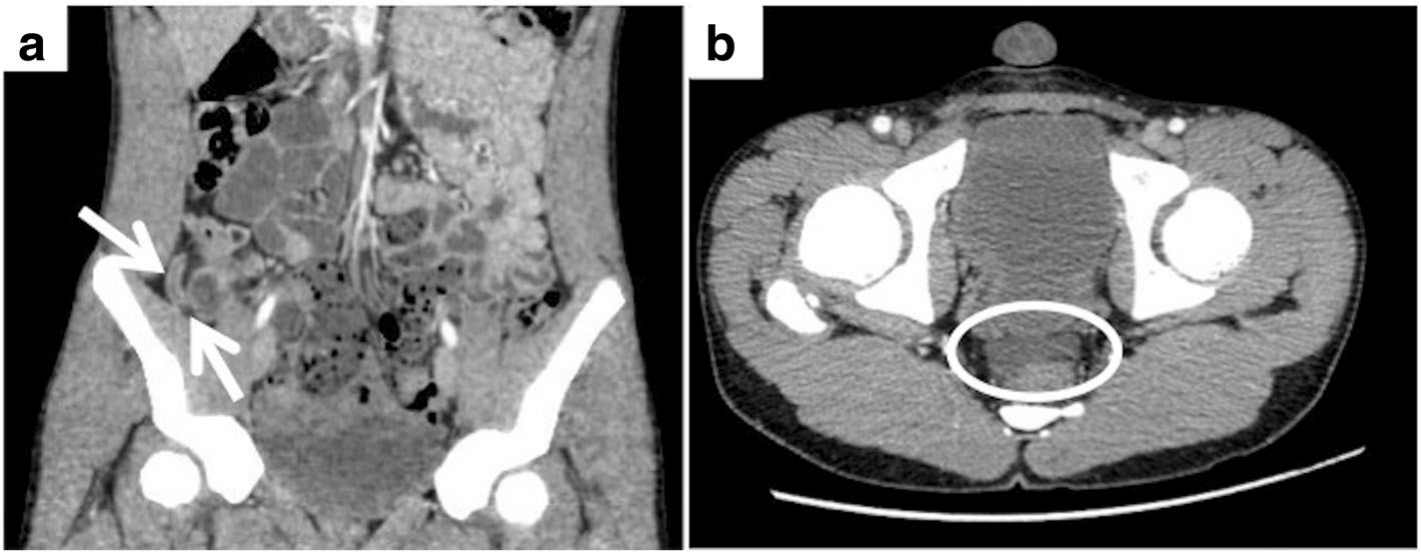
Enhanced computed tomography scan (CT) on admission. **a** CT shows a slightly enhanced and thickened appendiceal wall (arrow). Although the ileum is slightly dilated, intestinal obstruction cannot be detected. **b** CT also shows ascites at the recto-vesical pouch

A 14-year-old male was admitted to our hospital with right lower abdominal pain and vomiting. He had no history of abdominal surgery or trauma. Physical examination revealed deep tenderness at McBurney’s point without abdominal distension. A blood test on admission revealed slight leukocytosis (9840/μl) without elevation of the C-reactive protein level (0.01 mg/dl). Enhanced computed tomography scan showed a slightly enhanced, thickened appendiceal wall (Fig. 1a). Although a slightly dilated ileum and ascites at the recto-vesical pouch were also observed (Fig. 1b), intestinal obstruction was not diagnosed by these imaging studies. These abnormalities were thought to be inflammatory changes due to appendicitis. The initial diagnosis was mild acute appendicitis based on the physical examination findings and blood test and imaging results, and appendectomy with small laparotomy was therefore planned. However, the patient’s abdominal pain was so severe that analgesics were completely ineffective; continuous vomiting was also observed. Additionally, the ascites at the recto-vesical pouch was unusual considering the mild appendicitis. We performed laparoscopic surgery to explore the abdominal cavity and obtain a definitive diagnosis.

**Fig. 2:**
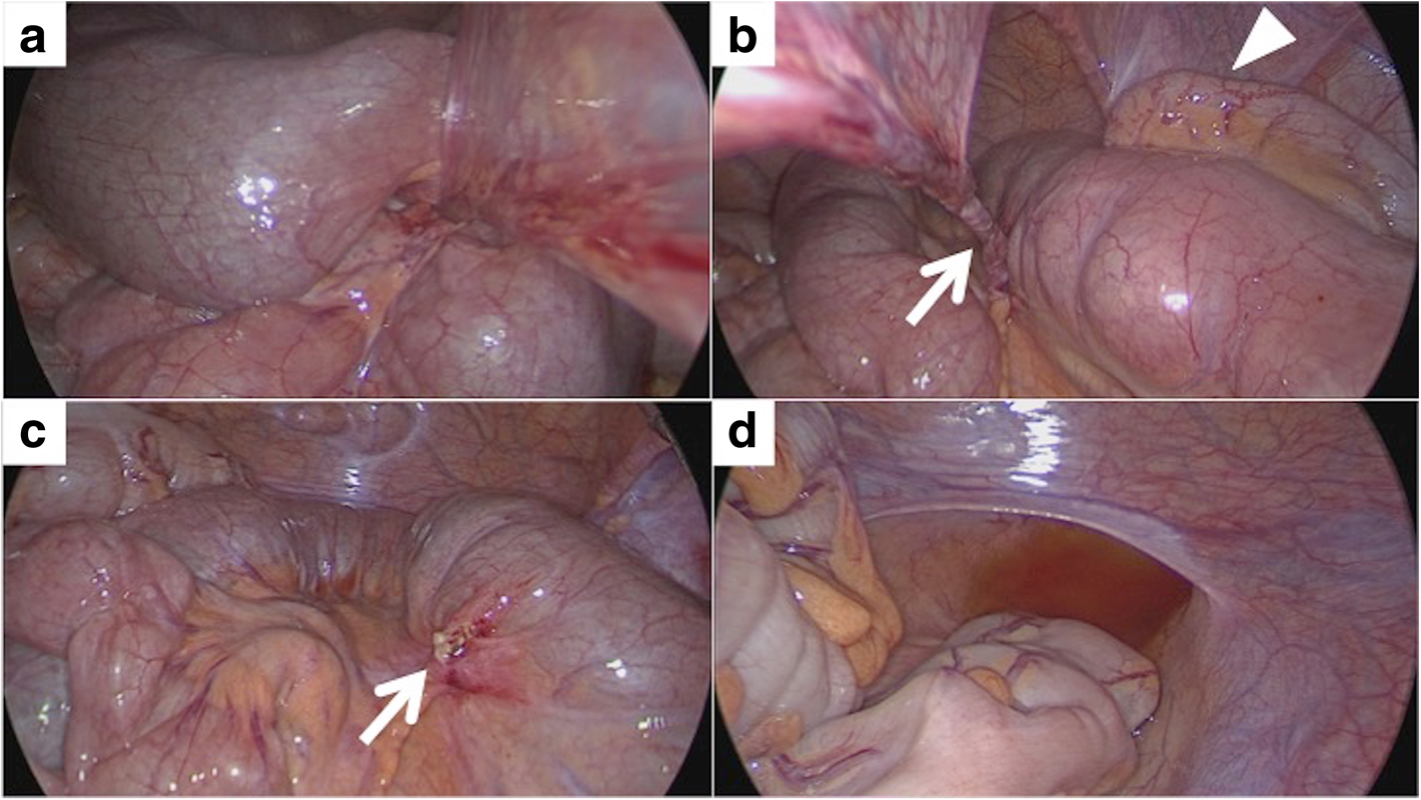
Intraoperative findings. **a** The ileum was trapped and strangulated by a band. **b** After reduction. The band was connecting the right medial umbilical fold to the ileal mesentery (arrow). Slight inflammatory change was observed on the surface of the appendix (arrowhead). **c** The band was connected to the ileal mesentery, 30 cm proximal to the ileocecal valve. Meckel’s diverticulum was not detected. The trapped ileum showed no ischemic change. **d** Serous ascites was observed at the recto-vesical pouch

Laparoscopic surgery with three trocars was performed (12-mm camera trocar in the infra-umbilical position and two 5-mm trocars in the left lower quadrant and lower median abdomen). Strangulated small bowel obstruction caused by trapping of ileal bowel loops by a band was observed (Fig. 2a). After reduction, the band was found to be connecting the right medial umbilical fold to the ileal mesentery (Fig. 2b, c) and was resected using laparoscopic coagulation shears. The band was connected to the ileal mesentery, 30 cm proximal to the ileocecal valve, and neither Meckel’s diverticulum nor ischemic change of the trapped ileum was detected (Fig. 2c). The appendix showed slight inflammatory change (Fig. 2b), and appendectomy was also performed. Serous ascites was found at the recto-vesical pouch (Fig. 2d) and was thought to be caused by strangulated small bowel obstruction. The patient was discharged without complications on postoperative day 4.

**Fig. 3:**
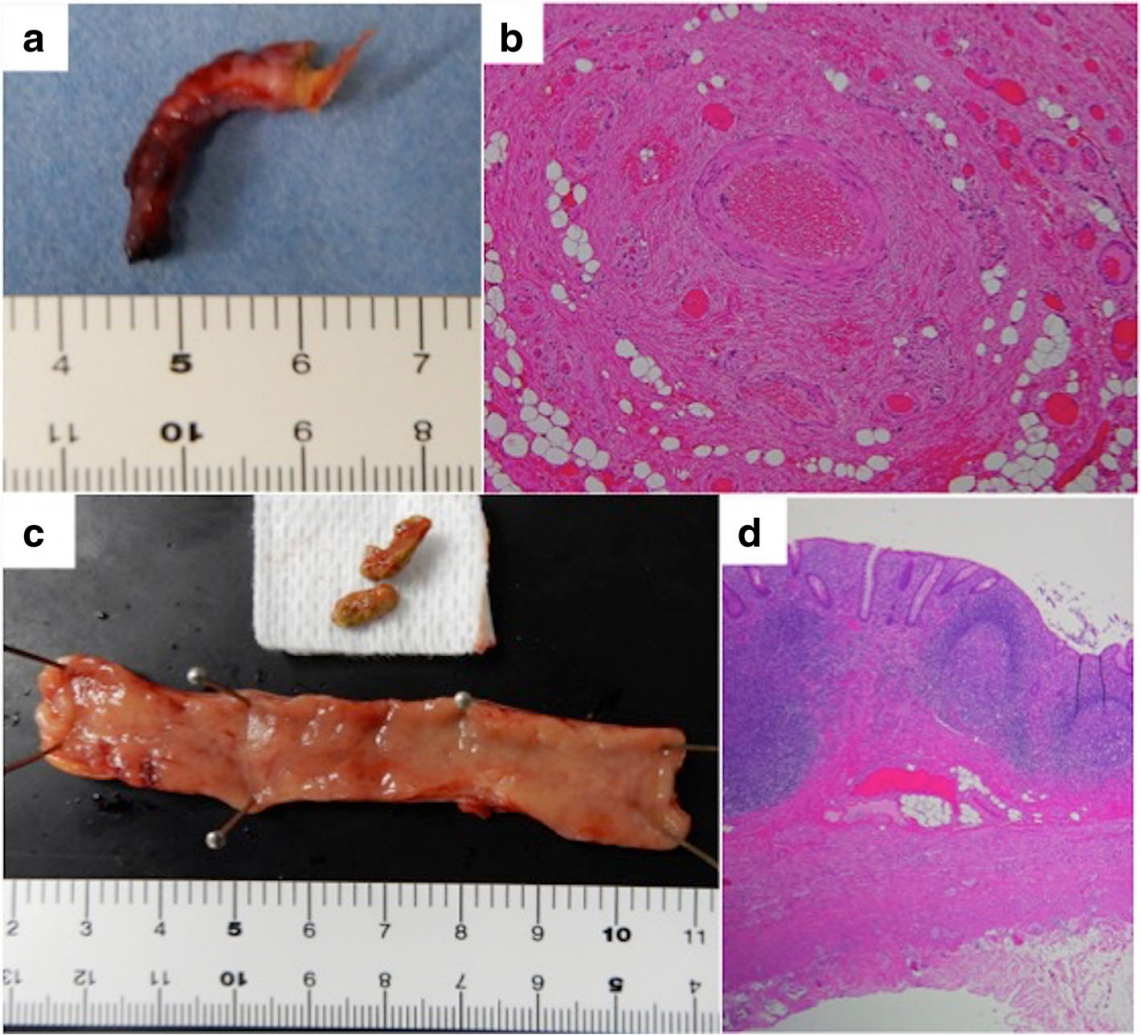
Macroscopic and pathological findings of the resected specimens. **a** Macroscopic findings of the band. **b** Microscopic examination revealed that the band consisted of blood vessels. **c** Macroscopic findings of the appendix. The appendiceal wall was slightly thickened, and fecal stones were found on the proximal side of the appendix. **d** Microscopic examination revealed diffuse infiltration of lymphocytes and eosinophils throughout the muscularis propria

Pathological examination revealed that the band consisted of blood vessels, and it was diagnosed as a vitelline vascular remnant (Fig. 3a, b). The macroscopic view of the resected appendix is shown in Fig. 3c. Fecal stones were found on the proximal side of the appendix, and the wall was slightly thickened. Pathological examination revealed diffuse infiltration of lymphocytes and eosinophils throughout the muscularis propria, and the patient was diagnosed with mild chronic appendicitis (Fig. 3d).

## Conclusions

Vitelline or omphalomesenteric duct remnants are rare congenital anomalies associated with the primitive yolk stalk. The vitelline duct is an embryologic communication between the primary yolk sac and the embryonic midgut, which gradually narrows and finally closes by week of 9 gestation [1–8]. Incomplete closure of the vitelline duct on the antimesenteric side can persist as a variety of anomalies. Meckel’s diverticulum is the most common, but others include vitelline duct fistula, sinus tract, cysts, and fibrous cords from the intestine to the umbilicus [7, 8]. Vitelline arteries originate from the primitive dorsal aorta and travel with the vitelline duct, and its remnant persists as fibrous bands covered by peritoneum. Rutherford et al. [3] have found that the vitelline vascular remnant coursed along the side of mesentery and insert into Meckel’s diverticulum or the posterior wall at the umbilicus. Furthermore, a vitelline vascular remnant sometimes occurs without Meckel’s diverticulum [9]. Thus, the patient was diagnosed with vitelline vascular remnant.

A vitelline duct remnant can result in several complications such as gastrointestinal tract bleeding, intestinal obstruction, abdominal pain, and umbilical discharge [1–8]. A vitelline vascular remnant may lead to intestinal infarction or necrosis resulting either from volvulus around the band or entrapment of the intestine [5]. Vane et al. [6] evaluated 217 children with vitelline duct remnants and found that 85 (40%) of the patients were symptomatic, whereas the remaining 132 were incidentally found to have an asymptomatic Meckel’s diverticulum. Complications of a vitelline duct remnant usually occur in young patients, and the presence of the remnant is associated with a high rate of mortality [1, 4]. Thus, surgical resection of an incidentally found vitelline duct or vascular remnant should be considered at the time of surgery for other conditions [6].

A vitelline duct remnant often contains heterotopic mucosa which can result in some clinical manifestations such as infection and bleeding [10]. Furthermore, several cases of malignant neoplasms arising in a vitelline duct remnant have been reported [11, 12]. Complete surgical resection of vitelline duct remnant is curative [10], and thus, segmental resection of the ileum might sometimes be required. Although deciding appropriate treatment intraoperatively might be difficult, especially for symptomatic vitelline duct remnant patients, careful intraoperative and histological examination is needed to achieve complete resection.

In the present patient, the preoperative diagnosis was mild appendicitis. Although this diagnosis was pathologically confirmed, the main symptom might have been caused by the intestinal obstruction secondary to the vitelline vascular remnant. Although suspected appendicitis with acute lower abdominal pain is one of the most common indications for emergency surgery in young patients, achieving an accurate preoperative diagnosis remains difficult despite recent improvements in imaging technology. Actually, Aitken [1] reported that the preoperative diagnosis was appendicitis in 28 of 73 patients with a symptomatic vitelline duct remnant. Laparoscopic surgery for a patient with acute abdomen might be useful for obtaining a definitive diagnosis during the operation, leading to appropriate treatment. Giri et al. [13] reported that additional laparoscopy during open appendectomy in patients with a normal-looking appendix helped to make a precise diagnosis and administer appropriate treatment. In the present patient, laparoscopic surgery allowed for a definitive diagnosis of intestinal obstruction and appropriate treatment. Laparoscopic surgery might be considered when a patient exhibits unusual symptoms, as in the present patient.

Several cases of vitelline duct remnants successfully treated with laparoscopic surgery were recently reported [14, 15]. Although laparoscopic surgery is reportedly useful for intestinal obstruction [16, 17], careful consideration is needed in patients with intestinal obstruction because of the surgical difficulty caused by the limited working space in the abdominal cavity. Although our patient was successfully treated with laparoscopic surgery because his abdomen was not distended, severe abdominal distention or peritoneal signs suggesting peritonitis, which necessitates prompt completion of the procedure, might be contraindications to laparoscopic surgery.

In conclusion, this is believed to be the first case report of the simultaneous occurrence of intestinal obstruction caused by a vitelline vascular remnant and appendicitis successfully treated with laparoscopic surgery. Achieving a definitive preoperative diagnosis of a vitelline vascular remnant might be difficult, especially in patients with coexisting appendicitis. When young patients demonstrate unusual symptoms, surgeons should consider the possibility of a vitelline vascular remnant, and laparoscopic surgery should be considered because it allows for simultaneous diagnosis and treatment.
